# Transgender Children: From Controversy to Dialogue

**DOI:** 10.1080/00797308.2021.1975432

**Published:** 2021-10-22

**Authors:** Jordan Osserman, Hannah Wallerstein

**Affiliations:** a Department of Psychosocial Studies, Birkbeck, University of London; b Austen Riggs Center

**Keywords:** Gender, transgender, trans kids, puberty blockers, development, countertransference

## Abstract

This paper introduces the topic and unique format of the section that follows, on psychoanalytic work with transgender children. We first review the apparent impasse that characterizes our field regarding clinical work with gender diverse kids, as well as the reasons we pursued a live dialog to push thinking forward. Then, we outline the structure of the entire section, in which four contributors offer short essays, followed by a transcribed and edited version of the dialog we facilitated, which uses these essays as a starting point. We conclude with reflections on some of the themes that arise in the dialog, and implications for all of us who work in the arena of gender and young people.

Seven years ago, this journal published a special section entitled *Transgender Children: Conundrums and Controversies*. Psychoanalytic clinicians were invited to address the question of children who identify with a gender different from their sex assigned at birth. There was a particular focus on the increasing availability of medical interventions for such children, such as the medical suspension of puberty, to “buy time” for further exploration and potential gender transition before an unwanted natal puberty sets in.

At the time of publication, transgender children were already a subject of fierce debate within and outside the psychoanalytic community. One might have hoped that attempts such as this journal’s previous special section to bring differing clinical perspectives together may have produced some degree of consensus within the field of psychoanalysis. On the contrary, just as society has become even more polarized around the subject of “trans,” and trans kids, so too the conjunction of “psychoanalysis,” “trans,” and “children,” seems to have reached an impasse.

Consider the following recent, pertinent example: In December 2020, the United Kingdom’s High Court of Justice (2020) made international headlines with its ruling on *Bell v Tavistock*. The case concerned the question of whether young people were sufficiently competent to consent to taking puberty blockers as a treatment for gender dysphoria. In a decision that challenged the current mainstream medical standards for transgender care, which support the use of such drugs in cases of severe and persistent gender dysphoria (WPATH 2012), the court ruled that the possibility of such consent was “highly unlikely” for children under the age of 16 years. This led the UK’s only public clinic for gender-related care – the Gender Identity Development Service (GIDS), part of the Tavistock and Portman NHS Foundation Trust – to immediately halt the process for under 16 year olds to begin puberty suppression treatment. The full implications of the ruling remain uncertain, with a subsequent ruling enabling parents to consent to puberty blockers on behalf of their children, and an appeal to *Bell v Tavistock* underway (see GIDS 2021; Middleton 2021).^1^

The key complainant in the case was Keisha Bell, who began transitioning to male at the age of 16, came to regret this choice, and subsequently de-transitioned, now identifying as female.^2^ Among those who publicly championed Keisha Bell’s case was psychiatrist and psychoanalyst David Bell (no familial relation), past president of the British Psychoanalytical Society and recently retired staff governor of the Tavistock and Portman NHS Foundation Trust. David Bell became known to the wider public after he wrote a highly critical report of GIDS that was leaked to the press, based on confidential conversations he held with a number of concerned staff in the service (Cooke 2021; Doward 2018). He has since become a prominent spokesperson, alongside other psychoanalysts, psychodynamic therapists, and “gender critical” activists, against mainstream approaches for children who wish to undergo gender transition (see Brunskell-Evans and Moore 2018, 2019). In their view, the field of mental health is failing young people and their families by too readily offering “affirmative” pharmacological solutions, foreclosing alternative outcomes that would be made possible through longer lasting, better resourced, and more exploratory talking therapies that retain explicit distance from views articulated by advocates for trans rights. “Gender services tend toward a damaging simplification,” writes Bell, partly because “most do not regard themselves as psychoanalytic services, and in some major services it is a small minority who have any substantial psychotherapeutic experience” (Bell 2020, 1032– 1033).

David Bell’s work has been especially focused on under-addressed social factors he believes contribute to the current rise in referrals for gender dysphoria, particularly among natal females, including an internalization of societal misogyny, the treatment of patients as consumers, and the influence of social media (Bell 2020). In a similar vein, in a public resignation letter following Bell’s report, Marcus Evans, a longstanding Tavistock governor and psychoanalyst, criticized “faddish” notions of “innate gender identity” which, he argued, prevents clinicians at GIDS and elsewhere from interrogating the meanings encoded in gender (Evans 2020). Bell, Evans, and other gender critical psychotherapists share the view that problems of identity require symbolic working through, and that interventions into the reality of the body are often mistaken, potentially psychotic solutions (Brunskell-Evans and Moore 2019).

While these statements may suggest that psychoanalysis is consolidating a position on trans kids and how to care for them, we are also witnessing a growth of psychoanalytic literature that supports young people’s access to gender related medical interventions, and is alert to the countertransferential reactions that subtend analysts’ anxieties around the subject (Farley and Kennedy 2020; Gozlan 2018; Saketopoulou 2020; Wiggins 2020). “Some psychic conflicts cannot be resolved in the psychic realm, requiring action,” writes Avgi Sakteopoulou in a recent debate with David Bell (2020, 1025). She has coined the concept “massive gender trauma” (2014) to describe the consequences that can attend to a child’s experience of misrecognition in the face of gender dysphoria. Here, it should be remembered that Diane Ehrensaft, a founder of the “gender affirmative” approach to care, whose contribution framed the debate of the previous *Conundrums and Controversies* section, is herself psychoanalytically trained, and draws on Winnicott in her coinage of the term “True Gender Self” (Ehrensaft 2015, 2009, 2021). Ehrensaft argues that the “gender affirmative” model is “particularly suited to psychoanalytic child therapy, where through listening, playing, mirroring, observing, relating, and interpreting, we strive to get to the heart of the gender matter” (2021, 77). As Saketopoulou has underlined (2020), this literature is often in conversation with other fields such as feminist, queer and trans studies, and foregrounds trans people’s own testimonies as an important point of reference (see especially Rose 2016).

Interestingly, much of this more trans-affirmative psychoanalytic work has been produced on North American soil, perhaps reflective of a divide between American psychoanalysts’ eagerness to accommodate progressive societal developments, and a British tradition that, with its emphasis on internal object relations, is more cautious regarding the complex symbolic processes that underlie seemingly liberatory external changes (see Bonfatto and Crasnow 2018; Lemma 2013, 2016, 2018). Alessandra Lemma, for example, while supportive of young people’s access to medical transition, cautions more enthusiastic advocates that “it remains our ethical responsibility to help our patients to also consider that even so-called creative acts come at significant costs and have multi-layered meanings that are not immediately accessible to our consciousness and that this requires processing (i.e. time) in order to make informed choices” (Lemma 2018, 1101).

That the UK controversy concerns a gender clinic located within the Tavistock, a psychodynamically oriented public mental health institution, is also noteworthy. Indeed GIDS has been contending with the tensions that characterize our field regarding the treatment of trans children since its inception in 1989. Founder and former director Domenico Di Ceglie was psychoanalytically trained and oriented the clinic around a Kleinian model of what he calls “atypical gender identity organization” (2018). This model holds open the possibility both that a transgender life is a liveable one and, when appropriate, ought to be medically facilitated; but also, that non-normative gender expression may communicate something other than a wish for transition. Currently GIDS’s multidisciplinary staff have diverse conceptualizations of gender identity, and often draw from contemporary psychiatric and psychological research, rather than psychoanalytic discourses (Costa, Carmichael, and Colizzi 2016); however, Di Ceglie’s psychodynamic approach continues to inform the literature clinicians at the service publish about their work (Bonfatto and Crasnow 2018; Wren 2014). In addition to allegations such as David Bell’s that GIDS does not allow enough time for exploratory work, the service has also been criticized for being too cautious in its approach to medical intervention, emphasizing the importance of exploration and waiting at the expense of patients in need (Green 2008). GIDS clinicians write about the need to hold on to complexity and curiosity amidst the intense pressures they experience from all sides, and the need to consider their interventions in relation to other systems and cultural contexts more generally (Bonfatto and Crasnow 2018; Wren 2019). “Practising ethically in such a service is not helpfully reduced to a single event, a treatment decision aimed at achieving the morally ‘right’ outcome,” writes GIDS’s former Consultant Psychologist Bernadette Wren (2019, 203), “but an extended process in time.”

Thus it is apparent that, contrary to a simple division between “gender affirmative” and “gender critical” camps, psychodynamically oriented clinicians exhibit a range of views on whether, when, and in what form children ought to be supported in physical and social gender transition (see Lament 2014 for a helpful overview). A recent intervention in the Lacanian field by Jacques Allain Miller (2021) sounded a particularly ambiguous note, at once accusing trans activists of making bad faith critiques of his school’s alleged transphobia, and of his own followers failing to listen to trans people in the particular way Freud listened to his hysteric patients.

This combination of extreme political polarization, alongside a range of sometimes incompatible clinical perspectives, can be bewildering to the contemporary clinician hoping to work effectively and ethically in this arena. It can create a destabilizing sensation of stuck-ness. In a way, the popular anxieties accompanying the prescription of puberty blockers – of children being placed in suspended time – reproduce themselves via the “trans debate” itself seeming suspended in time. Against this, psychoanalysis emerges as a discipline uniquely attuned to the ethical imperative to stay with a problem whose solution is not readily forthcoming (see Baraitser 2017). “As psychoanalysts,” writes Roger Litten in his “Introduction to a Conversation on the Trans Question” (2021), “we cannot afford to remain silent, to remain shut off from these questions as if they were no concern of ours … We either find ways to address this new configuration seriously, with all the attention it deserves … or we face up to the prospect of the disappearance of psychoanalysis as a viable clinical discourse.”

While statistical medicine has responded to anxieties surrounding gender in young people by attempting to predict which children are likely to “persist” versus “desist” in a trans identity – to diagnose whether a child is “really” trans, through behavioral criteria based on numerical averages (see Steensma et al. 2013; Temple Newhook et al. 2018) – this special section, functioning as “part II” of the original 2014 section on transgender children, is based on a wager that something different is required from our field. Namely, that we cannot sidestep the fact that every instance of childhood gender dysphoria involves a person with a unique subjectivity and emotional and relational life; and therefore, we cannot produce a “rulebook” for how to work in this arena, but rather must rely on the imperfect art of “thinking in cases” (Forrester 2017), over time and in dialogue. Indeed, if one thing might be identified in common across current psychodynamic perspectives – as we will see in the contributions that follow – it is a concern for allowing an individual’s suffering to be heard in its specificity, so that an adequate solution can be molded out of the impasses that we all face in relation to questions of sex and being.

## Contributors and format

Thus, without putting excessive faith in psychoanalysis’s ability to resolve larger social antagonisms, we both felt that psychoanalytic writers and practitioners *could* think collaboratively about how to bring to bear their unique attention to questions of desire, relationality, and the unconscious, onto the clinical situation of young people navigating the deeply personal, yet also universal challenge of gendered selfhood. While the previous special section began to explore this question, it was limited to individual contributors responding in written form. In contrast, we believe that a live exchange, where clinical and theoretical standpoints and differences can be hashed out in real time, holds open the possibility to move the conversation forward, perhaps alleviating the sense of “stuckness” and producing some shared coordinates from which a psychoanalytic way of working with trans and gender questioning children might proceed. Not unlike a clinical encounter, a live exchange requires participants to think on their feet, challenging the easy comforts of maintaining one’s pre-articulated perspective, and, we hope, enabling genuinely new insights to co-constructively emerge.

To this end, we developed the following structure: four clinicians and scholars who have written about trans issues from a generally psychoanalytic frame were asked to compose short, condensed pieces, stating what mattered most to them about clinical work with trans children and adolescents, as springboards for a live discussion. We offered a series of possible prompts, underlining a variety of areas that seem to us to require further thought. Our hope in giving contributors wide reach but limited space was to facilitate distilled thinking about the aspects of clinical work with trans children most salient to each contributor. We then circulated the pieces and used them to ground a 2–3 hour dialog we facilitated over Zoom, which was then transcribed, and is here published in edited form along with the preliminary short essays.

In selecting contributors, we prioritized having trans studies and child-developmental perspectives represented, alongside a generally psychoanalytic frame, as we believe all three orientations bring important insights to work with gender diverse children.^3^ These frameworks are not always compatible, and we were particularly interested in finding a way to speak across their differences. Additionally, we felt it was important to include those with direct experience of trans identity, in order to bring a first-hand perspective that could help articulate and identify concerns that non-trans contributors might overlook (on ethical research concerning members of oppressed groups, see Charlton 1998; Vincent 2018).

It is worth noting that in our search for contributors, we came across several scholars and clinicians who felt unable to participate, either due to their perception of the views of others we had asked, or due to their sense of vulnerability in their professional lives. This we think reflects the level of polarization, and the real barriers to speaking across differences in the field right now. We had especially hoped to find a UK participant working in the NHS with trans kids, but were ultimately unsuccessful, due both to the tense political situation that has placed clinicians under heightened scrutiny, as well as the workload strain brought on by chronic underfunding of gender-related healthcare. We are thus all the more grateful for and impressed by the courage of the contributors to this section, for their willingness to engage despite the risks and demands on their time.

The contributors are as follows: Dr. Oren Gozlan is a clinical psychologist and psychoanalyst in private practice in Toronto. He is chair of the Gender and Sexuality Committee of the International Forum for Psychoanalytic Education, and has written extensively on the topic of transgender. His book “Transexuality and the Art of Transitioning: A Lacanian Approach” won the American Academy and Board of Psychoanalysis’ book prize. His most recent article addresses fantasies surrounding the treatment of trans children. Gozlan’s short essay for this volume continues his exploration of countertransferential dynamics at play in adult concerns for trans children, with a particular focus on anxieties about change and regret.

Dr. Laurel Silber is a child analyst in private practice in Philadelphia. She is past president of the Philadelphia Society for Psychoanalytic Psychology and Philadelphia Center for Psychoanalytic Education, adjunct faculty at Widener’s Institute of Graduate Clinical Psychology and faculty at the Institute of Relational Psychoanalysis of Philadelphia. She is a long-time advocate for child therapy and has written on childism, the intergenerational transmission of trauma, and more recently on gender expression and variance in children. Silber makes her own plea in this volume for attending to adult anxieties that may cloud the work with trans and gender variant children, reading these anxieties in the seeming lack of concern, or hasty support for action, that forecloses the space for play and curiosity.

Dr. Eve Watson is a psychoanalyst and university lecturer in Dublin, Ireland. She specializes in sexuality studies, and is currently course director of the Freud Lacan Institute as well as editor of Lacunaue, the APPI International Journal for Lacanian Psychoanalysis. Watson has published extensively on psychoanalysis, sexuality, and film. She is coauthor of *Clinical Encounters in Sexuality: Psychoanalytic Practice and Queer Theory* with Dr. Noreen Giffney, and author of a book chapter on the treatment of trans children in *Lacanian Psychoanalysis with Babies, Children and Adolescents*. Her preliminary piece for this section focuses on the importance of approaching gender as a “query” and not an endpoint, the value of time, and the oppressive force of idealizing trends within mental health care.

Lastly, Dr. Tobias Wiggins is an assistant professor of Women’s and Gender Studies at Athabasca University. His research and writing focuses on transgender mental health, queer, and trans-visual culture, clinical transphobia, accessible community-based wellness and psychoanalysis. Wiggins coordinates the University Certificate in Counseling Women, an interdisciplinary program applying feminist theory to the practice of counseling and, among other advocacy projects, is a current member of the Alberta Trans Health Network, a collaborative group of healthcare providers, researchers, and community-based working in trans, non-binary, and Two-Spirit health in Alberta. Most recently Wiggins has written about perverse countertransference in relation to trans subjects. In this volume, he introduces Elisabeth Young–Bruehl’s notion of “childism” to read the particular position of the trans child in debates about transgender more broadly.

## Present and absent perspectives

Looking at the contributions as a whole, a few characteristics stand out. First, we are very pleased to gather a relatively international group, albeit Anglo-American, and readers will notice the different contexts each contributor is speaking from. Some of the contributors, for instance, are in a position to advocate for or determine a child’s access to medical interventions, while others work separately from the medical establishment. Second, only one of our contributors, Silber, works primarily with children, as opposed to adolescents and adults. While this was partly due to contributor availability, it also highlights the dearth of contemporary child-centered analytic writing about trans experience, perhaps due to it being an especially contested arena. Finally, although we set out to include participants from a wide range of theoretical orientations, our conversation has a Lacanian emphasis (albeit not exclusively so, with Silber representing a relational perspective). We do not think this is simply a coincidence, but rather, reflective of the fact that Lacanian thought has played an outsized role in contemporary, English language psychoanalytic writing on trans (see Carlson 2010; Cavanagh 2016; Gherovici 2010, 2017; Gozlan 2014; Osserman 2017; Shepherdson 2000).

This is a curious development (especially given the marginalization of Lacanian clinical practice within the Anglophone world) for which various explanations may be offered. Lacan has, until recently, had a stronger foothold within academia compared to other psychoanalytic theorists, and the university is also a key site for transgender politics.^4^ A kind of circulation from the academy to the clinic characterizes psychoanalytic discussions of gender, and trans in particular, and Lacan becomes a seemingly inevitable companion to this itinerary. On a more theoretical level, the Lacanian emphasis on the symbolic order’s “denaturalization” of gender and sexuality – the ways that language and culture tear us away from animal instinct – has been a longtime resource for feminist, queer and trans theory that seeks to dethrone the seeming naturalness and inevitability of gender and sexual normativity (Dean 2000; Mitchell 2000; Rose 2005, 2016; Salamon 2010). Lacan challenges his followers to reject both biological determinism and voluntarist fantasies of unlimited possibility (as sometimes appears in more euphoric writing about gender), and this speaks to a problem at the heart of clinical work with gender diversity: how can a person’s sense of their gender appear both radically at odds with their sexual anatomy, yet also largely outside of their voluntary control or manipulation?^5^

Despite this Lacanian emphasis, we believe that the problems explored within these pages are relevant to psychotherapeutic practitioners of all orientations, and are grateful to our contributors for engaging across theoretical schools in accessible language.

## I’m a boy/”I a boy”

To orient readers to some of the tensions that fill the following pages, we thought to highlight a particularly charged, and also fertile, moment in our dialog, that occurs around the sharing of a clinical vignette. Silber speaks of a young child, assigned female at birth, who lost their father under tragic circumstances, and who began to repeat the phrase, “I a boy.” Silber uses this example to point to the enigmatic, relational ways that children play out questions of gender, and the need for such play to be collaboratively and creatively translated. Wiggins expresses concern for the way such narratives risk reinforcing the transphobic notion that beneath gender nonconformity lies an unresolved trauma that, if worked through, will invariably return the subject to a cisgender position.

While we will let our readers judge for themselves how this exchange plays out, here we draw attention to an interesting slippage we noticed when we revisited the exchange. While Silber quoted the child uttering the ambiguous phrase “I a boy,” Wiggins’ remarks referenced the phrase “I’m a boy.” This is not to criticize Wiggins for making a “mistake,” but rather to attend to the kind of linguistic slippage that characterizes the dialectical movement of psychoanalytic thought. For, these two phrases, which differ phonetically by the mere presence of the sound “m,” offer a world of difference in potential meaning, and speak to fundamental tensions around the subject of trans kids.

“I a boy” brings into view primary process aspects of gender: ungrammatical and enigmatic, it invokes the child’s early, tumultuous acquisition of language, and struggle to distinguish between subject and object as it negotiates its place within its relational world (or in Lacanian terms, the discourse of the Other). The gendered binary girl/boy, and its possible relations to mother/father, emerges here as an indeterminate, affective charge, in need of translation and containment.

By contrast, “I’m a boy” makes sense. It is a familiar grammatical phrase that puts the question of identity in (seemingly) clear and straightforward terms. While such a statement may still concern family relations and the enigmas of coming into language, it is also a fully formed assertion of self that articulates a boundary (I am x, not y). Where the former statement requires translation in order to be understood, here there is a choice point, regarding inquiring further or taking the self-assertion as an end point. When spoken by a child assigned female at birth, the statement calls forth a series of questions that may be more or less anxiety provoking (depending on the recipient of the message) yet that nevertheless belong to a certain framework of interpretation. Whether treated as a statement of incipient trans identity, a passing fancy, a bit of “gender creativity,” or even, for some, an indicator of pathology, the phrase participates in a social discourse whose contours are relatively well understood.

As such, the opposition “I a boy”/”I’m a boy” gives form to crucial tensions that emerge in our discussion: first, between how psychoanalysis understands the process of subject development for everyone versus how it speaks more specifically to contemporary trans discourse, and second, between the particularities of childhood and more general ways of thinking about gender experience. For instance, is identity conceptualized as something enigmatic and related to split subjecthood, or as something knowable, communicable, and able to be politicized? And should a child’s gendered statement be heard similarly to an adult’s? In which ways, and in which ways not? There are no simple answers to these questions. Often, when people speak from one side of these tensions, they can see the other side as alternately transphobic, or unable/unwilling to interrogate subjectivity outside of identarian terms. Yet we believe each side offers something vital to the conversation, as becomes apparent in the following pages. Before turning our readers over to that, we will mark three more specific areas of tension that strike us in the engagements that follow.

## The dependency relationship

One glaring difference in work with children and with adults is the dependency that defines childhood. While certainly no adult is completely independent, the dependencies of childhood are pervasive, asymmetrical, and legally defined. Winnicott’s much cited comment that there is “no such thing as a baby” (1964, 88) may well be expanded to the entire period of childhood, albeit to progressively lessening degrees, as ideally development moves the child from a state of total to increasingly partial dependence on others.

This has implications for the treatment of trans children, which differ from that of adults. Namely, providers and parents have a duty to protect children, to some degree, and to be more active participants in decision-making. But what, exactly, trans children need to be protected from, is a matter of contention. Our contributors take this up in different ways. Watson and Silber focus on the protection of a time for exploration, or a play space in which further elaboration can occur. Gozlan and Wiggins focus more on the protection of children from adult biases and projections that can distort listening. Additionally, in different ways, all contributors call our attention to the complicated affects and desires that can creep into a felt need to “protect the children,” raising the question of how one sorts out the difference between the misuse of a child for adult needs, and responsible caregiving that takes stock of the actual dependency relationship.

Ideas about protection are especially salient in discussions on the use of medical interventions such as puberty blockers with young adolescents. On one side of the debate, there is an argument that children should be protected from the responsibility of making decisions involving bodily intervention. Here a young or emerging adolescent is understood to be too young to comprehend the risks and consequences of puberty blockers. However, trans children are seen as in need of protection from puberty itself, and the associated distress. Where in the former argument waiting is imbued with a protective function, here doing nothing is seen as the larger risk, and puberty blockers as a relatively low-risk option with high gains. As we reflected on these tensions as they play out in the dialog, we came to think that where one lands has at least in part to do with beliefs and values each of us carry regarding the significance of bodily interventions and the role of biology in psychic development. The literature on puberty blockers as a treatment for gender dysphoria in adolescents is relatively new, with current treatment protocols showing generally positive psychological outcomes (if you subscribe to the findings and presuppositions of statistical psychology, which are often questioned by psychoanalysts), alongside indications of some possible risk to bone density and fertility (Mahfouda et al. 2017; Rew et al. 2021; Turban et al. 2020). How do we think about these kinds of risks and uncertainties? Are they serious enough to warrant abstaining, or are the benefits (such as the relief of distress or the possibility of a better esthetic outcome) large enough to outweigh them? And when it comes to considering the psychological impact of delaying puberty, how does one think about the relationship between psychic life and biological processes? We felt that participants’ different views and concerns on these questions reflected larger ideological differences and value systems that cannot be neatly sorted into “progressive” or “conservative.”

## Gender and meaning

Another area of tension concerns the question “why?,” or the linking of gender to meanings that move beyond it. This is central to the moment between Silber and Wiggins referenced earlier, where Wiggins pushes back on Silber’s elaboration of a gendered expression in relation to trauma. Both highlight important aspects to the question of gender and meaning. Silber advocates for the need to think about all that may be represented by a gendered statement, in addition to its face value. Here the searching for “why” becomes a means for expanded listening, to help catch more of a child’s experience and facilitate its movement into representation. Silber’s focus on translating a child’s statements is a useful counterpoint to the reverence for unknowability that Gozlan articulates, as it points to the prior need to experience one’s expressions as understandable or digestible. This process of coming-to-know and to experience one’s thoughts as knowable is arguably necessary in order to eventually face the limits to what can be known.

While sensitive to the dangers of “truncating” the emotional complexity of gender and transition, Wiggins, on the other hand, alerts us to the ways such an exploratory process can, and does, become perverted. This is a subtle, but important point, that hypotheses or interpretations offered under the guise of further exploration can be made in order to push a patient toward a more normative gender. Gozlan describes this as the “Russian Doll” approach, where there is a search for the layers of meaning underlying a gender expression, with the assumption that after the layers have been uncovered the gender variance will disappear. Taken together, these points remind us of the fragility of a space for actual curiosity, and the important clinical tension between respecting a patient’s statements and inviting elaboration.

## Affirmation vs. neutrality

A final area of tension concerns the gender affirmative model of care, which has gained widespread support and is presented by many in the mental health field as the treatment of choice when working with gender non-conforming children. For those unfamiliar, Wiggins offers a clear and succinct definition of the affirmative model as one that “asks the child about their gender identity, believing in the authentic face-value of their gendered assertions, and supports requests for physical and social transition.” Such direct support appears to most contributors in stark contrast to psychoanalytic ideas of neutrality and the discipline’s refusal to take any statement at “face value,” a tension that plays out productively in the dialog that follows.

A consensus becomes clear among contributors regarding the implicit support of referring to patients by the pronouns or names they ask us to. However, when it comes to explicit support for medical treatment, or direct affirmation of patients’ statements about their identities, more nuanced differences emerge. Watson criticizes what she sees as the ready-made quality of the affirmative model, in that offering immediate support for a patient’s statements implies that the statements can and have been quickly understood. Such quick support here communicates that the face value, or socially accepted meaning of a statement is all that need be looked at, which does not allow the singularity of the patient’s speech to be addressed or supported. Wiggins is concerned that when immediate support is withheld, exactly the space Watson imagines being opened up will be shut down, especially given the larger social context of failed mirroring and outright refusal that permeates the lives of trans people. Together, Watson and Wiggins evoke the tension between attending to the radical singularity of patients while also taking account of the shared social and political phenomena in which we live.

This tension between affirmation and neutrality left us thinking about a third term, acceptance, which relates to but differs from both. While one could define neutrality as the (full) acceptance of all aspects of a patient, the latter term underlines the inviting element in the stance. This is to highlight that what can pass for neutral can in reality be skepticism, anxiety, or even blatant refusal – just think of the variety of communications that can be delivered in the phrase “no comment” or the general atmosphere thereof. But acceptance of a patient’s statements also differs from affirmation, in that it refers to a more passive, rather than active process. Acceptance is of the order of receptivity; something is taken in, in order to be thought about and worked with. Affirmation, on the other hand, is outwardly directed; something external is shored up. Where accepting a patient’s statements is a first step toward further thinking, affirming them is the solidification of thoughts that have already occurred. When it comes to the psychoanalytic task of facilitating personal discovery, it seems the former is the more apt, as it is an open, and so positive, engagement that also does not claim authority over or reify what is ultimately someone else’s process. But actual acceptance of what our patients bring us, perhaps akin to actual curiosity, is much easier to speak about than implement, especially within an arena as charged as gender. We believe that each contributor exhibits a commitment to precisely this treacherous, albeit necessary task. Together, they carve out a way of working with trans children that is deeply thoughtful and respectful, paving a way forward for all of us.

## Footnotes

1 The decision has had reverberations across Europe, with Sweden subsequently basing a decision to halt under 16s’ access to puberty blockers on the Tavistock ruling (Parsons 2021; see also The Economist 2021).

2 It is noteworthy that this decision, which applies to children under the age of sixteen, relied on the evidence of someone who began her medical transition after that age.

3 Gender diversity refers broadly to gender identities, expressions or roles that differ from the norms associated with one’s assigned sex. The term was developed as an alternative to “gender non-conforming,” which can be seen as stigmatizing. While no term is without problems, we use the terms interchangeably here in accordance with their broader use. For more information on terms, see Key Concepts In Understanding Gender Diversity and Sexual Orientation Among Students (APA 2015).

4 More recent academic engagement with the work of relational psychoanalysts such as Jessica Benjamin, as well as Winnicott and Klein, suggests that Lacan’s influence here may be waning (e.g. Ahmed 2006; Allen 2020; Bowker and Buzby 2017; Butler 2021).Interestingly, this seems concurrent with a rise in Anglo-American clinicians engaging with Lacanian ideas and approaches (at least in our anecdotal experience).

5 Until recently, Lacanian analysts, like many others, tended to conceive of the demand for transition as a psychotic “solution” likely to produce further psychic turmoil if actualized. This view has been significantly challenged and complicated in recent years due, in no small part, to the pioneering work of Lacanian analyst Patricia Gherovici (2010, 2017).
